# Impacts of Climate Change on Inequities in Child Health

**DOI:** 10.3390/children1030461

**Published:** 2014-12-03

**Authors:** Charmian M. Bennett, Sharon Friel

**Affiliations:** 1 National Centre for Epidemiology and Population Health, Australian National University, Acton ACT 0200, Australia; 2 Regulatory Institutions Network, Australian National University, Acton ACT 0200, Australia; E-Mail: Sharon.Friel@anu.edu.au

**Keywords:** child health, health inequity, climate change, social determinants, vulnerability, under-nutrition, poverty, vector-borne disease, heat stress, extreme weather events

## Abstract

This paper addresses an often overlooked aspect of climate change impacts on child health: the amplification of existing child health inequities by climate change. Although the effects of climate change on child health will likely be negative, the distribution of these impacts across populations will be uneven. The burden of climate change-related ill-health will fall heavily on the world’s poorest and socially-disadvantaged children, who already have poor survival rates and low life expectancies due to issues including poverty, endemic disease, undernutrition, inadequate living conditions and socio-economic disadvantage. Climate change will exacerbate these existing inequities to disproportionately affect disadvantaged children. We discuss heat stress, extreme weather events, vector-borne diseases and undernutrition as exemplars of the complex interactions between climate change and inequities in child health.

## 1. Introduction

The impacts of climate change on health will vary significantly across the globe due to differences in the underlying health status of populations, and the uneven distribution of social, economic and cultural factors that affect different population group’s exposure and capacity to respond and adapt to environmental hazards. Climate change will have a disproportionate effect on the most vulnerable and disadvantaged populations—those whose lives and livelihoods are heavily dependent on the environment, who already experience high disease burdens that are affected by environmental hazards, and who live in physical and social conditions that are vulnerable to environmental pressures.

Children are especially vulnerable. Their developing bodies are sensitive to environmental hazards (such as heatwaves and the spread of infectious diseases), and damage experienced during these critical years can have life-long impacts. Within and between countries, there are already dramatic differences in the life chances for a child depending on the circumstances in which they are born, grow, work and age. This reflects the unequal distribution of many essential determinants of health, such as access to clean water, adequate sanitation systems, a nutritious diet, safe housing and gender equality. Climate change will act as an amplifier of these existing inequities to have a disproportionate effect on the health and wellbeing of children across the globe. The world’s poorest and socially-disadvantaged children will bear the greatest burden of climate change-related ill-health.

In this paper, we discuss how social inequities already affect child health outcomes, and explore how climate change will act on these existing inequities to disproportionately affect child health around the world. We discuss four examples of major climate-related risks to child health (heat stress, extreme weather events, vector-borne diseases and undernutrition), and show how these child health threats are exacerbated by inequities in social, economic and cultural factors. We conclude by discussing the strong synergies between what is needed to provide every child with the chance to live a long, healthy and productive life, and the adaptations required to respond to current and future climate risks in general.

## 2. Existing Inequities in Child Health

There have been massive improvements in child survival and life expectancies over the last half century, which are the result of not only technological and medical change, but also social factors. For example, access to education encourages new family structures in which women and children are allocated greater priority in terms of care and allocation of food within the household [1]. Educated mothers also begin to move beyond a fatalistic acceptance of high childhood mortality amongst their offspring towards the implementation of simple health-promoting practices, such as keeping a clean home and boiling water before use [2].

However, there are still substantial inequities in child health outcomes that are inextricably linked to social and economic gradients of advantage and disadvantage within and between countries. Unequal economic growth, unequal conditions of daily living (especially access to food, water, shelter and basic healthcare) and the suppression of human rights (particularly by gender and ethnicity) have led to established inequities in life chances.

More than 200 million children under 5 years of age in developing countries do not reach their development potential due to poverty, malnutrition and poor health, which disrupts their cognitive, physical and social-emotional development [3]. Compared to better-off families, children from poor families are more likely to be exposed to pathogenic agents (due to inadequate sanitation, housing and water supplies); once exposed, they are more likely to become ill (due to lower resistance to infection and poor access to preventive interventions); once ill, they are less likely to have access to health care and effective treatments. This contributes to a life trajectory of poor health, lack of readiness for school, poor academic performance, subsequent low incomes, high fertility rates and inability to provide optimal care for their children, thus perpetuating the intergenerational cycle of poverty. Poorer children are therefore more likely than their peers to die during childhood, or be undernourished, chronically ill and experience sub-optimal health throughout their lives [3,4,5,6].

## 3. The Impact of Climate Change on Environmental Health Hazards in Childhood

Up to two-thirds of preventable illness and death from environmental hazards is experienced by children [7,8,9], and the burden is predominantly in those aged under 5 years [10]. Children are more vulnerable than adults to changes in their environment, due to their small physical size, physiological and cognitive immaturity, and their dependence on caregivers for safety and protection [11].

Children have distinct physiologies and exposure profiles that mean climate-sensitive diseases place an undue burden on the youngest members of society. Compared to adults, children have much higher exposures to environmental hazards. For example, children tend to spend more time outdoors, where they can be exposed to high temperatures and where disease vectors such as rodents, mosquitos and ticks are found. Children require more water (by weight) than adults, so their exposure to water-borne pathogens is much higher. Diarrheal diseases cause dehydration in children much faster than in adults, and children are more likely to develop severe infection and experience complications during recovery due to their small body size and their developing immune systems which provide little natural immunity or resistance [8,12].

Importantly, there are distinct “windows of vulnerability” during gestation and early childhood, when critical biological systems such as the immune and central nervous system are developing. Maternal undernutrition, infection and illness at these critical times can cause devastating and life-long damage, including physical stunting, neurological impairments and immune dysfunction [7,13]. In addition, their young age means that more years of that child’s life will be lived in a climate-changed future, thus their potential lifetime exposures to environmental hazards will be greater than today’s adults [7].

## 4. Pathways between Inequities in Child Health Outcomes and the Impacts of Climate Change on Environmental Hazards

**Figure 1:**
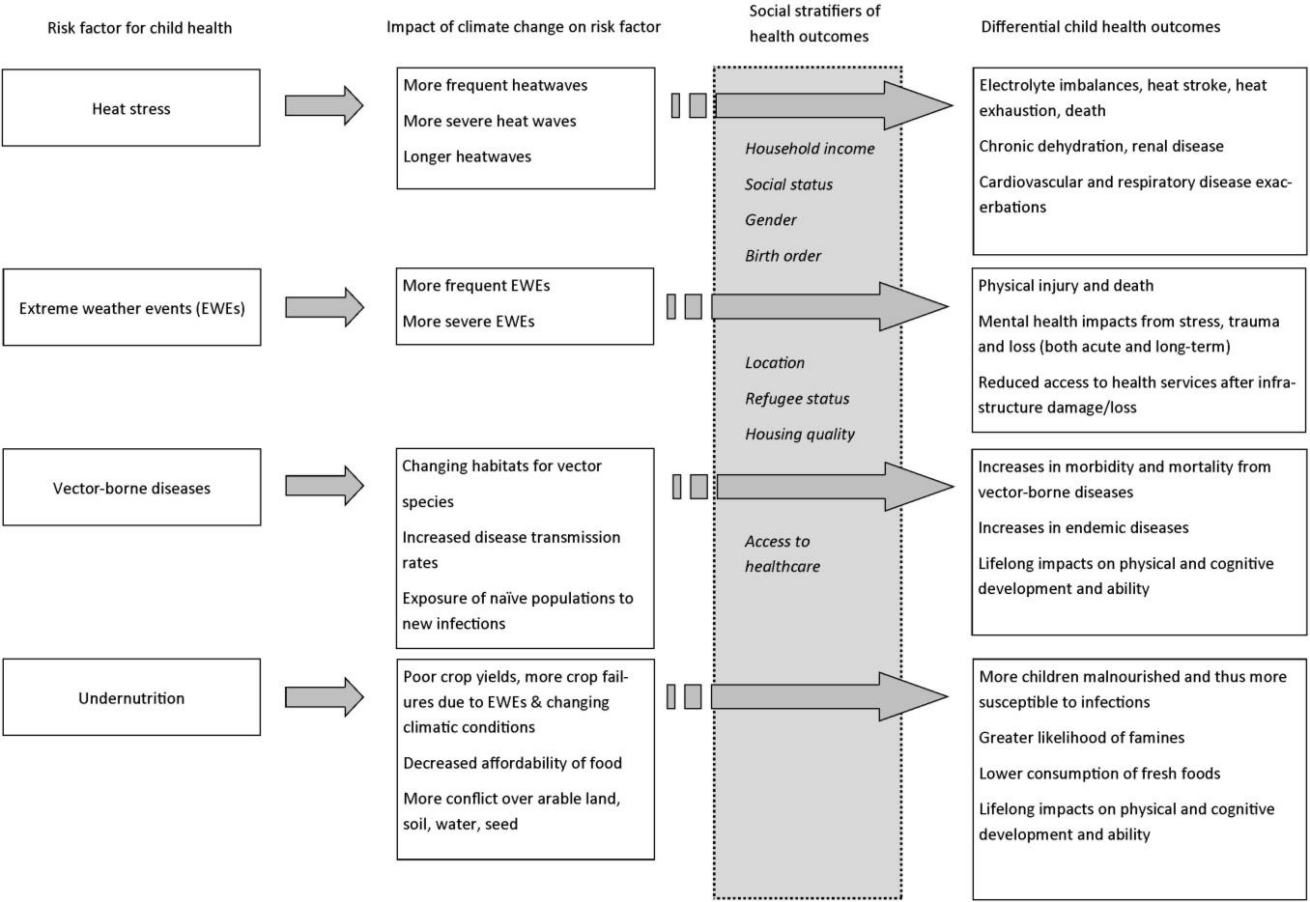
Pathways between selected environmental hazards, climate change, and social conditions that lead to differential health outcomes for children.

Inequities in child health outcomes may be exacerbated by climate-related changes in the frequency and severity of environmental hazards, especially heat stress, extreme weather events, vector-borne diseases and undernutrition. In all of these examples, children have greater vulnerability to adverse health outcomes compared to other population groups [14] and existing inequities will amplify these health risks to children. The pathways between these particular environmental health hazards, climate change, and issues of inequity that lead to differential health outcomes for children are illustrated in Figure 1.

### 4.1. Heat Stress

Infants and young children are at high risk of ill-health and death related to extreme heat exposure. Children tend to spend more time outside, with more vigorous activity, than adults, thus placing them at increased risk of dehydration, electrolyte imbalances, heat stroke and heat exhaustion [7,14,15]. Increases in daily maximum temperatures have been associated with increases in emergency department visits for heat-related illness, electrolyte imbalances, fever and gastroenteritis in children under 6 years in the US and Australia [7,16,17] and increases in mortality rates with rising temperatures have been observed in those under 15 years in Brazil [8]. The risk of preterm birth and stillbirth have been shown to increase with rising daily temperatures [18,19] and renal disease has been linked to hyperthermia in children [7].

The health risks from a heatwave are strongly mediated by social advantage or disadvantage. During a period of extreme heat, a child from a high income family in a developed setting can retreat to insulated buildings, with electricity to run air conditioning and clean, cold water on tap. In contrast, a child living in an urban slum, with no running water or electricity and poorly made shelter, has no refuge from the heat and no way to artificially cool themselves. Slums and poor neighbourhoods also tend to be devoid of vegetation, which offers some protection from the heat, so the slums are likely to also be hotter than more developed urban areas nearby [20]. Children in this setting are thus at much higher risk for heat-related illness and death [21,22].

### 4.2. Extreme Weather Events

Climate change is expected to bring more frequent and more severe extreme weather events, such as storms, cyclones, floods and droughts. Children are particularly vulnerable to these hazards as they are heavily dependent on their caregivers for safety and protection during such events. When the immediate threats to health and safety have subsided, there are invariably lingering effects related to material losses (including homes and possessions), infrastructure damage (such as loss of health services or access to relief supplies), the displacement of large numbers of people (which may be permanent), loss of livelihoods and slow economic recovery.

Disadvantaged communities have little ability to cope with disasters, especially a series of events in close succession. They also have little ability to recover and rebuild afterwards. This was clearly illustrated in New Orleans following Hurricane Katrina, where the poorest residents lived in the lowest-lying suburbs, which were flooded and destroyed. Most of the emergency evacuation plans relied on the assumption that affected residents owned a car, had money to fill the tank with petrol, and had a safe place to go away from the storm. The residents of the worst-affected suburbs had few of these [23]. Later, it was clearly shown that the majority of deaths occurred in these disadvantaged populations who were unable to escape the approaching storm and did not have the resources to protect themselves or their property.

Children are particularly at risk of injury and death from storms and floods [14]. Water-borne diseases are common during and after floods, as water supplies, sanitation and sewage systems are damaged or inundated, cooking facilities are disrupted and people are crowded into emergency shelters where outbreaks of infectious diseases can run rampant in unsanitary conditions. Children’s immature immune systems are less able to resist infection, and their small body size means that diarrheal diseases can cause children to become dangerously dehydrated very quickly. In developed countries, outbreaks of infectious disease after disasters are usually short-lived and relatively mild [14], but in developing countries, the same outbreak superimposed on socio-economic disadvantage and entrenched poverty can be deadly. For example, in Nepal, the risk of flood-related mortality significantly increases with low socio-economic status and poor quality housing [24]. The flood mortality rates are also higher for girls than boys [24].

Many post-disaster emergency housing settlements are over-crowded, unsanitary and impoverished, and plagued by outbreaks of infectious diseases, episodes of violent conflict over scarce resources and persistent food insecurity. Basic life essentials (such as safe water and adequate shelter) are hard to obtain and livelihoods disappear. Many children become separated from their families and caregivers and are at greater risk of sexual assault, abuse and neglect [25]. In a climate-changed future with more frequent and more severe extreme weather events, and in a context of widening health inequities within and between countries, these unsafe and unhealthy environments will be where growing numbers of children may spend their formative years [8].

Meeting the physical survival needs of children during a disaster is not sufficient to ensure that their experience of the disaster does not continue to impair their mental, social and emotional development. Children are particularly vulnerable to emotional trauma from sudden changes in their daily lives, social networks and sense of security [9]. Mental and emotional distress is commonly reported in children and adolescents following natural disasters, including post-traumatic stress disorders, sleep disturbance, aggressive behaviour, depression and anxiety, and substance abuse [14,26,27,28]. But adverse mental health impacts of stress and trauma may not be immediately apparent, and can persist for extended periods post-disaster [29,30]. There is already clear evidence that traumatic events during the childhood years can have lasting effects on mental health and cognitive development [26,29,30]. The risks are particularly high for children without strong social support networks (such as those in displaced or fragmented families) [25,31,32], for those with a family history of psychological distress [30], for those with low family incomes [30] and for those who experience subsequent traumatic events during childhood [29]. Parental stress also has an important impact on child health outcomes following a disaster. Studies have shown that rates of child maltreatment, abuse and inflicted traumatic brain injuries increase in the 6 months following natural disasters [33,34], which is thought to be related to parental stress associated with the loss of resources and/or livelihoods, and the disruption of social support structures.

### 4.3. Vector-Borne Diseases

Climate change will alter the ecological context in which disease hosts, vectors and parasites breed, develop and transmit disease [35]. The distribution of many infectious disease vectors, including rodents, ticks and mosquitoes, is limited by the availability of suitable habitats and they are highly sensitive to changes in temperature, humidity and rainfall.

Changes in temperature and rainfall regimes have implications for the spread of many infectious diseases. Most obviously, climate change will alter the distribution of potential habitats for vector species and it is predicted that the habitat ranges of mosquito species that transmit malaria, dengue fever and yellow fever, and the tick species that transmit encephalitis and Lyme Disease, will expand as a result of rising global temperatures [14,36,37,38]. The spread of vector species will thus expose immunologically naïve populations to new infectious diseases [39].

Children are prone to vector-borne diseases because they spend more time outdoors and they are closer to the ground, where vector species commonly gather [9]. Their small body size and developing immune systems means that children are more susceptible to vector-borne diseases than adults. Children are also more likely to experience more severe disease, experience more complications and are less likely to have a complete recovery than adults, especially for malaria, dengue fever, encephalitis and Lyme Disease [7,9]. For the most advantaged populations, vector-borne disease may be avoidable and treatable, but the synergistic effects of endemic vector-borne disease, lifelong poverty, persistent malnutrition and lack of preventive health measures in disadvantaged populations can be deadly.

Poverty often coincides with regions of endemic infectious disease. Heavy burdens of endemic disease have significant impacts on productivity and health of the workforce, which leads to reduced household incomes and exacerbates economic disadvantage in those who can least afford it [40]. In addition, many endemic vector-borne diseases, including malaria, meningitis and tuberculosis, can have life-long impacts on a child’s physical and/or cognitive development, thus childhood infection can dramatically reduce the chances for a healthy and productive adult life.

Children in poor families are likely to have poor immunity to infection as a result of persistent under-nutrition. Once sick, they lack access to healthcare, are unable to purchase medicines or obtain adequate nutrition to recover completely, leading to sub-optimal health outcomes and greater chances of complications during recovery [5,41]. Poor families also lack the capacity to utilize preventive healthcare, such as sleeping under bednets, using insect repellants and completing childhood vaccination schedules. In contrast, a child in a more advantaged situation will likely have better immunity as a result of better nutrition and completed vaccinations, so will be less likely to be infected in the first place. In addition, they will have better access to healthcare if they do get sick, be more effectively treated and thus recover fully with minimal lasting impacts.

The economic costs of a vector-borne disease epidemic are strongly mediated by socio-economic status. Epidemics in populations of low socio-economic status have substantial household costs, including foregone income from sick adults and those caring for sick children, the costs of healthcare and medicines, and missed schooling. Subsistence farmers and their families suffer disproportionately from the loss of income as well as loss of food from their land.

Malaria is one of a number of key developmental risk factors that contribute to pervasive poverty in sub-Saharan Africa. An estimated 400 million children under the age of 5 are infected with malaria each year [42]. In addition to high mortality rates (around 20%), childhood malaria is associated with acute and long-term neurological, cognitive and physical impairments in around 25% of survivors, including losses in motor function, co-ordination, speech, hearing and vision, as well as psychosis, seizures and epilepsy [42,43,44,45]. Many of these impairments become increasingly apparent over time as the child ages and faces more complex cognitive and physical demands. Childhood infections can therefore have major impacts on health and well-being in adult life, with survivors burdened with physical and cognitive deficits that lead to a less productive working life, whilst increasing the demands on caregivers, and thus affecting both the strength and the productivity of the future workforce [40].

In malaria-endemic regions, many children build up some immunity as a result of repeated infections in the first 10 years of life [44]. The greatest changes in risk will occur at the current margins of the malaria parasite distribution as rising temperatures allow the mosquito vector to spread into higher altitudes and the desert fringes where malaria is not endemic and the populations therefore have little or no immunity [39,44,46]. Low population immunity heightens the risk of severe disease in children, and such epidemics are characterized by much higher mortality rates (up to 10 times higher) than disease in malaria-endemic regions [47]. In either situation, the persistent effects of malaria infection extend the burden of disease far beyond the conventional measures of morbidity and mortality [42].

El Niño/Southern Oscillation (ENSO) events have been examined as a proxy for a climate-altered future, as they often alter weather patterns in the direction of a warmer climate for periods of several years at a time. ENSO events have been linked to increases in hospital admissions for diarrheal disease in Peruvian children, where a 5 °C increase above normal temperatures equated to a 200% increase in hospital admissions [48]. Millions of children in impoverished situations die from preventable diarrheal diseases every year, mostly in developing countries, and future rises in temperature as a result of climate change could have devastating effects.

### 4.4. Undernutrition

The expected impacts of climate change on the distribution of rainfall and rising temperatures, as well as increases in the frequency of heat waves, floods and droughts, will destabilize agricultural production in many regions, and exacerbate the continued degradation and loss of agricultural lands globally due to desertification, chemical contamination and urban sprawl. This will, in turn, lead to decreasing crop yields, greater chances of crop failure, disruption of growth and harvesting cycles, and thus, changes in food availability and price.

Africa and southern Asia already experience heavy childhood disease burdens from entrenched poverty, endemic infectious disease, malnutrition and inadequate sanitation. These regions are also expected to bear the brunt of climate-related decreases in crop yields, as dryland and semi-arid, non-irrigated agriculture predominates and many staple food crops are already near their maximum temperature tolerance [49]. Subsistence farmers and pastoral communities will suffer the most, as their livelihoods, and those of their families, depend on a reliable crop yield.

The impact of climate change on agricultural yields and productivity is likely to have profound impacts on child health outcomes, especially in regions with heavy dependence on subsistence lifestyles and where undernutrition and malnutrition is already prevalent. Children require more food per unit mass than adults [13], and most of the world’s hungry are children. Malnutrition already kills more than 3 million children every year (mostly in southeast Asia and sub-Saharan Africa). A further 178 million children suffer from malnutrition, and one third of all children under five in developing countries are chronically malnourished or stunted [50]. Nutrient-deficient diets, undernutrition and malnutrition weaken overall health, and are closely tied to unsanitary living conditions and socio-economic disadvantage [51,52]. Endemic disease and undernutrition also fuel a vicious self-perpetuating cycle of disadvantage: the risk of infection (and complications from infection) is much higher if the child is malnourished, and the risk of malnourishment is greater if the child already has an infectious disease [7,8]. This cycle of poor health leads to permanent physical, cognitive and psychological impairments that will have profound impacts on the ability of that child to lead a healthy and productive adult life, including caring for children of their own. Maternal malnutrition results in decreased birthweights and restricted intrauterine growth, and those children are then much more likely to become shorter adults, receive less schooling and experience reduced economic productivity in adult life than their better nourished counter-parts [52,53].

The stress of food insecurity associated with future climate change may also amplify inequities in the distribution of food within a household. Gender inequalities in nutrition have been documented in Bangladesh [54] and India [55], with more than half of all severely malnourished children being female. Cultural norms may dictate that male children are more highly valued than female children, based on their future earning potential, social and religious roles in society, kinship structures, and their future ability to defend and support their family [56,57]. As a result, male children within the household may be prioritized over female children to receive food and water. Birth order may also affect food allocations within a household, especially in tough times, when families may discriminate against younger, more dependent children in favor of older, more productive children [55,56,58].

There is also a strong potential for climate change to alter nutrition and dietary habits among socially disadvantaged populations in more developed settings. The urban poor are especially vulnerable, as they already spend a large share of their income on food and non-agricultural households must purchase the majority of their food [32]. As agricultural yields decline or fail, the retail price of fresh foods will rise, putting them out of reach of many low-income household budgets, who may then turn to cheaper, more heavily processed, energy-dense foods instead. Local crop failures may also place increasing reliance on fresh foods from external sources (*i.e.*, imported food items)—the increased transport costs associated with food importation will be passed on to the consumer through price rises which will, again, place many fresh food items out of reach for many low income people. Climate change will thus markedly reduce the quantity, quality and affordability of food for disadvantaged populations in both rural and urban settings [59].

## 5. Conclusion: A Way Forward—Reducing Child Health Inequities to Reduce the Impacts of Climate Change on Child Health

The inherent biological vulnerabilities of children interact with the health risks associated with social disadvantage (especially poverty, class and gender) to amplify the risks to child health from climate change, both within and between populations and regions.

Protecting and improving child health in a climate-changed world demands that we pay attention to the existing inequities in child survival and development [60,61]. The existing social and economic gradients in child health outcomes will be amplified by the ability of different populations to protect themselves and their livelihoods in the face of climatic change. Those with more resources, power and influence will be better able to cope with climate-related hazards, to recover from damaging climatic events, and to safely relocate their families if needed. In contrast, the most vulnerable populations will bear the burden of rising morbidity and mortality associated with climate change, including severe storms and droughts, infectious disease epidemics and widespread food insecurity.

There are strong synergies between what children need to thrive and become healthy adults, and the adaptations that are required to reduce the risks from environmental disasters including climate change. Sustainable economic development, the reduction of poverty and wealth disparities, improvements in baseline health status, food security and education are fundamental, as well as early warning systems, immunization programmes, drinking water disinfection and resilient sanitation systems. For example, the Millennium Development Goals (MDGs) address both climate change and health by aiming to reduce the background rates of disease and child mortality, thus reducing the underlying vulnerability of many populations to adverse health impacts related to climate change. Similarly, climate change adaptation and mitigation strategies address the negative consequences of climate change by aiming to reduce human vulnerability to climate change impacts, as well as addressing the drivers of that change.

There is a need to reorient both health and climate change policies to reflect today’s knowledge about the social determinants of health and health inequities, alongside the causes and impacts of climate change on global health [59,62,63]. There are many positive health co-benefits that can accrue from climate change-related adaptation and mitigation activities across a range of sectors, such as improvements in physical health from more active (and low emission) transport methods, and improvements in local air quality with cleaner forms of energy generation [64]. Integrating social and health policies into climate change adaptation and mitigation policies is a “win-win” situation for both developed and developing countries, and offers a strong framework with which to gain political and policy-related support for effective adaptation and mitigation strategies that reduce the negative impacts of climate change on health.

A critical step in the global response to climate change is to address the existing inequities in fundamental health-sustaining resources. Widespread access to safe water, adequate shelter, reliable food supplies, resilient sanitation systems, treatment for endemic diseases and gender equality will help to ensure that future generations of children are less likely to be poorly educated, physically and cognitively impaired, stuck in a vicious cycle of poverty and poor health, and thus be unable to reach their full potential. These issues were historically confined to the developing world, but with the recent surges in massive urban slum settlements on the fringes of large cities around the world, these risk factors are now embedded in developed urban populations as well. Similarly, with increasing environmental pressures on rural communities in both developed and developing countries, these risks to child health inequities will not just be challenges in the developing world.
